# X‐linked adrenoleukodystrophy: Pathology, pathophysiology, diagnostic testing, newborn screening and therapies

**DOI:** 10.1002/jdn.10003

**Published:** 2020-01-26

**Authors:** Bela R. Turk, Christiane Theda, Ali Fatemi, Ann B. Moser

**Affiliations:** ^1^ Hugo W Moser Research Institute Kennedy Krieger Institute Baltimore MD USA; ^2^ Neonatal Services Royal Women's Hospital Murdoch Children's Research Institute and University of Melbourne Melbourne VIC Australia

**Keywords:** clinical trials, inflammation, newborn screening, therapy, very long‐chain fatty acids, X‐linked adrenoleukodystrophy

## Abstract

Adrenoleukodystrophy (ALD) is a rare X‐linked disease caused by a mutation of the peroxisomal *ABCD1* gene. This review summarizes our current understanding of the pathogenic cell‐ and tissue‐specific roles of lipid species in the context of experimental therapeutic strategies and provides an overview of critical historical developments, therapeutic trials and the advent of newborn screening in the USA. In ALD, very long‐chain fatty acid (VLCFA) chain length‐dependent dysregulation of endoplasmic reticulum stress and mitochondrial radical generating systems inducing cell death pathways has been shown, providing the rationale for therapeutic moiety‐specific VLCFA reduction and antioxidant strategies. The continuing increase in newborn screening programs and promising results from ongoing and recent therapeutic investigations provide hope for ALD.

**Abbreviations**
ALD: X‐linked adrenoleukodystrophy
AMN: adrenomyeloneuropathy
BBB: blood–brain barrier
C26:0‐LPC: C26:0‐lysophosphatidylcholine
CALD: cerebral adrenoleukodystrophy
CH25H: cholesterol 25‐hydroxylase
ELOVL1: gene encodes ELOVL Fatty Acid Elongase 1
ER: endoplasmic reticulum
FA: fatty acid
HSCT: hematopoietic stem cell transplantation
24‐HC: 24‐hydroxycholesterol
25‐HC: 25‐hydroxycholesterol
27‐HC: 27‐hydroxycholesterol
7KC: 7‐ketocholesterol
LC‐MS/MS: liquid chromatography mass spectrometry
MRI: magnetic resonance imaging
NAC: N‐acetyl cysteine
OPCs: oligodendrocyte precursor cells
PUFA: polyunsaturated fatty acids
ROS: reactive oxygen species
UPR: unfolded protein response
VLCFA: very long‐chain fatty acids

## 1 INTRODUCTION

X‐linked adrenoleukodystrophy, ALD, (MIM #300100) is the most common peroxisomal disorder affecting both males and females with an estimated birth incidence of about 1/14,700 (Bezman et al., 2001; Moser et al., 2016). ALD is caused by a mutation in the *ABCD1* gene which encodes a peroxisomal ATP‐binding cassette transporter for very long‐chain saturated fatty acids ≥ C22:0 (VLCFA) into the peroxisome for β‐oxidation (Kemp et al., 2001). As of April 3, 2019, there are more than 2,707 mutations of *ABCD1* of which 812 are non‐recurrent and 248 variants of unknown significance (https://adrenoleukodystrophy.info/mutations-and-variants-in-abcd1). There is no phenotype/genotype correlation (Kemp, Berger, & Aubourg, 2012).

Most men and women with ALD have a slowly progressive spinal cord disease, adrenomyeloneuropathy, AMN, men, typically beginning in their 30s, and women beginning postmenopausal (Engelen et al., 2014; Huffnagel, Dijkgraaf, et al., 2019a). However, 35%–40%of ALD males may develop a rapidly progressive inflammatory cerebral demyelination peaking in the ages 3–10 years of age. About 20% of adult males with AMN also develop cerebral disease that rapidly progresses to disability and death (van Geel, Bezman, Loes, Moser, & Raymond, 2001). Additionally, the adrenal glands are commonly affected with a lifetime risk of adrenal insufficiency of ~80% in ALD males (Huffnagel, Laheji, et al., 2019b).

Allogeneic hematopoietic stem cell transplantation, HSCT, can halt the cerebral demyelination if done early before neurological symptoms and before advanced brain disease occurs. Early diagnosis through family screening of at‐risk males, and as of December 2013 in the USA, newborn screening has provided hope for successful treatment for ALD (Raymond, Moser, & Fatemi, 2018). Adrenal insufficiency is rare in ALD females but early identification of adrenal disease will save the lives of ALD males who may succumb to adrenal crisis without stress hormone administration (Dubey et al., 2005; Huffnagel, Laheji, et al., 2019b; Kemp, Huffnagel, Linthorst, Wanders, & Engelen, 2016).

Lipids containing VLCFA accumulate in all tissues; however, the brain, spinal cord, adrenal cortex and the Leydig cells of the testis have the greatest increase of VLCFA. VLCFA are mainly esterified with cholesterol and glycerophospholipids, resulting in pathology (Johnson, Schaumburg, & Powers, 1976). Studies show that other lipids such as free cholesterol, the oxysterols, gangliosides and the plasmalogens may also contribute to the pathophysiology of ALD. (Igarashi, Belchis, & Suzuki, 1976b; Khan, Singh, & Singh, 2008; Nury et al., 2017; Powers, 2005).

Understanding how the accumulation of VLCFA leads to adrenal insufficiency, the rapid inflammatory brain disease in cerebral ALD (CALD) and the gradual loss of function in spinal cord disease are some of the topics of current research in ALD. There are clinical trials of promising therapies for the slowly progressive spinal cord disease; however, to date, there are no established effective treatments. ALD has no phenotype/genotype correlation, nor can the course of the disease be predicted based on the levels of VLCFA, thus the search for modifier gene(s) continues (Kemp et al., 2012).

## 2 HISTORY OF ALD

### 2.1 Defining the phenotype

The first case of cerebral ALD was published by Haberfeld and Spieler in 1910. They described a boy who had been well until the age of 3 or 4 years when he was noted to be hyperpigmented. At the age of 6 and 1/2 years, he became disturbed, lost speech, the ability to walk, and died at 7 years. Postmortem examination showed adrenal atrophy and extensive demyelination with perivascular accumulation of lymphocytes and plasma cells at the leading edge of the myelin lesion (Haberfeld & Spieler, 1910). Schilder described several cases in 1912, 1913, 1924 and thus at that time ALD became known as Schilder's disease (Schilder, 1912, 1913, 1924). Simmerling and Creutzfeld published a case of ALD in 1923 (Siemerling & Creutzfeldt, 1923). Fanconi et al., in 1963, proposed X‐linked inheritance based on the analysis of 10 reported cases (Fanconi, Prader, Isler, Luethy, & Siebenmann, 1963). Michael Blaw introduced the term *adrenoleukodystrophy*, ALD (Blaw, 1970). In 1976, Budka et al. and Griffin et al. separately reported a form of ALD that presented as a slowly progressive paraparesis in adults, now known as AMN (Budka, Sluga, & Heiss, 1976; Griffin, Goren, Schaumburg, Engel, & Loriaux, 1977).

### 2.2 Discovery of lipid and fatty acid abnormalities

The pathological observations in ALD adrenal cortex, Schwann cells, testis and postmortem brain by Jim Powers, Herb Schaumburg and Anne Johnson led to the identification of the abnormal inclusions as cholesterol esters containing an excess of VLCFA (Johnson et al., 1976; Powers & Schaumberg, 1974a; Powers & Schaumburg, 1973, 1974b). The serendipitous and key demonstration of increased VLCFA content in brain cholesterol esters was made by M. Igarashi, a visiting scholar in the laboratory of Kunihiko Suzuki at Albert Einstein College of Medicine, New York. This was then confirmed by Yasuo Kishimoto, known for his expertise in brain fatty acid metabolism at the Eunice Kennedy Shriver Center for Mental Retardation, Waltham MA (Igarashi, Schaumburg, et al., 1976a; Moser, 2006). This discovery led to the diagnostic identification of ALD males and females by measurement of total lipid VLCFA in cultured skin fibroblasts, amniocytes and plasma in the early 1980s (Moser et al., 1981, 1980).

### 2.3 Inheritance and genetic defect

With this biochemical handle many ALD families were found including a family who was alssssso informative for the gene for glucose‐6‐phosphate dehydrogenase, G‐6‐PD, which maps to the terminal segment of the long arm of the X chromosome, Xq28. Linkage studies in this family showed that the gene for ALD also maps to Xq28 (Migeon et al., 1981). The locus was further defined in 1988 by Aubourg and Sack by studies in an ALD family where the ALD gene segregated with the gene for color blindness (Aubourg, Sack, & Moser, 1988). In 1993, the ALD gene, *ABCD1*, was identified by Mosser et al. (Mosser et al., 1993). The ABCD1 gene encodes the peroxisomal transmembrane protein, the ABCD1 protein, also known as ALDP. A member of the ATP‐binding cassette (ABC) transport family, the ABCD1 protein has the structure of an ABC half‐transporter and transports saturated straight‐chained VLCFA as CoA esters to the peroxisome where they are degraded via β‐oxidation (Kemp, Theodoulou, & Wanders, 2011).

## 3 CLINICAL FEATURES OF ALD

Clinical presentation of X‐linked ALD phenotypes are summarized in Table 1. These phenotypes are commonly used to describe cerebral, adrenal and spinal cords and peripheral nerve involvement. While no genotype–phenotype correlation is known, phenotype shift to deadly cerebral disease is typically only seen in homozygous individuals. (From Moser, Smith, Watkins, Powers, & Moser, 2001; Used with permission). Further details regarding clinical have been comprehensively published in multiple reviews (Dubey et al., 2005; Engelen et al., 2014; Huffnagel, Ballegoij, et al., 2019c; Huffnagel, Dijkgraaf, et al., 2019a; Huffnagel, Laheji, et al., 2019b; Moser et al., 2000; Raymond et al., 2018).

**Table 1:** ALD Phenotypes

ALD phenotypes		
	Presentation & pathology	Reported cumulative frequency and age of onset
*Phenotypes in males*		
Childhood cerebral (CCALD)	Progressive behavioral, cognitive and neurologic deficit often leading to total disability and death within 4 years of diagnosis. Pathologic hallmark is inflammatory cerebral demyelination	31%–35% Onset at 3–11 years of age
Adolescent cerebral	Presentation & pathology as in CCALD. Onset 11–21 years with somewhat slower progression than CCALD	4%–7% Onset 11–21 years of age
Adrenomyeloneuropathy (AMN)	Characterized by weakness, spasticity, pain, bladder & bowel dysfunction and impaired movement often resulting in assistive device or wheelchair use. Pathology includes slow progressive distal axonopathy with atrophy of the spinal cord, and peripheral neuropathy	Most adult males will develop AMN Onset typically starting in third‐fourth decade of life
Adult cerebral	Dementia, behavioral disturbances and focal neurologic deficits. Symptom progression may parallel CCALD, however, rate of progression is variable with rare self‐limiting cerebral demyelination termed “arrested‐cerebral disease.”	20% (van Geel et al., 2001)
Addison‐only	Primary adrenal involvement without apparent neurologic involvement. Most will continue to develop AMN	Common in childhood
Asymptomatic	Biochemical and gene abnormality without demonstrable adrenal or neurologic deficit. Detailed studies often show adrenal hypofunction or subtle signs of AMN on examination in adulthood	Common in childhood. 50% of asymptomatic develop AMN within 10 years
*Phenotypes in females*		
Asymptomatic	No evidence of adrenal or neurologic involvement	
Adrenomyeloneuropathy. Mild, moderate and severe	Symptomatology resembles AMN in men, albeit with later onset and a slower rate of progression	Increases with age. Estimates of 50% >40 and ca. 65% by 65
Cerebral involvement	Rare, reported in cases with confirmed and suspected X chromosomal inactivation	Few cases reported (Fatemi et al., 2003)
Addison's disease	Rare in females and does not precede AMN phenotype as seen in males	1%

## 4 BIOCHEMISTRY OF ALD

### 4.1 Peroxisomal metabolism of fatty acids in ALD

The *ABCD1* gene encodes the peroxisomal transmembrane protein, the *ABCD1* protein, also known as ALDP. A member of the ATP‐binding cassette (ABC) transport family, the *ABCD1* protein has the structure of an ABC half‐transporter and transports saturated straight‐chain VLCFA as CoA esters to the peroxisome where they are degraded via β‐oxidation. In ALD, the VLCFA accumulate in plasma and the cells of all tissues (Kemp et al., 2001). Only a small amount of VLCFAs are of dietary origin. The majority are the result of chain elongation by the ELOVL enzymes (Jakobsson, Westerberg, & Jacobsson, 2006; Tsuji, Sano, Ariga, & Miyatake, 1981). There are 7 ELOVL enzymes; however, *ELOVL1*, is the one responsible for the chain elongation of VLCFA (Ofman et al., 2010; Ohno et al., 2010).

Two other peroxisomal ABC transporters, *ABCD2* and *ABCD3* can assume overlapping functions with *ABCD1.* These are not mutated in ALD (Matsukawa et al., 2011). *ABCD1* and *ABCD2* are highly homologous and have an overlap in specificity for saturated and monounsaturated fatty acids. *ABCD2* expression is lacking in human fibroblasts; thus, the 10%–15% residual β‐oxidation in ALD is most likely due to *ABCD3* (Wiesinger, Eichler, & Berger, 2015; Wiesinger, Kunze, Regelsberger, Forss‐Petter, & Berger, 2013). Overexpression of either *ABCD2* or *ABCD3* in ALD fibroblasts was shown to be able to correct the biochemical defect (Kemp & Wanders, 2010; Kemp et al., 1998).

The saturated straight‐chain VLCFA are found in excess in ALD blood and most tissues esterified to glycerophospholipids, lysophospholipids, sphingolipids, acyl‐CoAs and acyl‐carnitines (Kemp & Wanders, 2010; Moser et al., 2001; van de Beek et al., 2016). In adrenal cortex, testis and in demyelinating brain there are large amounts of VLCFA esterified to cholesterol (Igarashi, Schaumburg, et al., 1976a; Theda, 1988; Theda, Moser, Powers, & Moser, 1992). Postmortem white matter from a patient with late onset ALD was obtained from different areas of the brain and classified according to the microscopic appearance as “intact” (occipital), “active” (posterior frontal) and “gliotic,” (frontal). The lipid analyses in three areas, showed distinct differences in the lipid composition. There was a marked increase in cholesterol esters containing VLCFA only in the “active” area. The C24:0 fatty acid content of the gangliosides was increased in ALD white matter from the “active” and the “gliotic” areas, and only slightly increased in the “intact” area when compared with control. The white matter ganglioside results confirmed the results reported by Igarashi, Belchis, et al. (1976b). The total phospholipids were increased in all ALD white matter samples when compared with control white matter and the galactolipids were decreased. The most striking finding was increased VLCFA, with a C26:0 fatty 17‐fold increase compared with control, in the phosphatidylcholine from the “intact” and the “active” white matter samples (Table 2 adapted from Theda et al., 1992 used with permission). These lipid analyses demonstrate that the increase in the phosphatidylcholine VLCFA precedes the onset of demyelination. Microcalorimetric studies have shown that the C26:0 excess disrupts membrane stability (Ho, Moser, Kishimoto, & Hamilton, 1995). The VLCFA modified phosphatidylcholine in the myelin membrane may result in changes in the structural integrity of myelin and lead to immunological mediated destruction of myelin that is characteristic of cerebral ALD.

**Table 2:** Lipid Composition of Control and ALD White Matter Samples

Water content, total lipids and lipid composition of control and adrenoleukodystrophy white matter samples	Control	ALD intact	ALD active	ALD gliotic
Water content (%)	73	65	75	89
Total lipids (% dry weight)	54.9	51.5	44.4	17
Free cholesterol (% of total lipids)	29.7	25.2	3.9	25.3^a^
Cholesterol ester (% of total lipids)	Trace	0.5	31	1.3^a^
Phospholipids (% of total lipids)	33.7	42.2	41.8	41.3^a^
Galactolipids (% of total lipids)	36.6	31.3	22.9	4^a^
Phosphatidylcholine (VLCFA as % total fatty acid)	1.57	10.36	16.66	
Phosphatidylethanolamine (VLCFA as % total fatty acid)	0.12	1.82	0.72	
Phosphatidylserine (VLCFA as % total fatty acid)	2.49	0.73	1.08	
Phosphatidylinositol (VLCFA as % total fatty acid)	1.05	2.68	3.21	
Gangliosides (C24:0 fatty acid as % total fatty acids)	2.95	4.3	10.22	14.64

a Triglycerides and free fatty acids: 28.1% of total lipids.

### 4.2 The role of cholesterol metabolism in the pathophysiology of ALD

Interestingly, the organs most affected by a deficiency of *ABCD1*, brain and adrenal, have the highest content of cholesterol in the body. While some investigations into the involvement of cholesterol metabolism in ALD have been made, we propose that cholesterol transport dysfunction may play a pathogenic role in oxidative response and inflammatory‐mediated processes.

#### 4.2.1 Cholesterol metabolism in brain

As lipoprotein‐bound cholesterol from the circulation cannot cross the blood–brain barrier, BBB, the majority of brain cholesterol is synthesized in the brain with 70% stored in myelin as free cholesterol with a very slow turnover (half‐life of approximately 5 years) and the rest in the plasma membranes of neurons (10%) and glia cells (20%) that turnover more rapidly (half‐life of 5–6 months) (Dietschy, 2009; Petrov, Kasimov, & Zefirov, 2016; Pfrieger & Ungerer, 2011). A surplus of cholesterol in neurons and other cells is stored as esters, thus about 1% of brain cholesterol in the normal adult brain is cytoplasmic cholesterol esters in lipid droplets formed by increased acyl‐CoA cholesterol acyltransferase 1 gene expression (ACAT1) in response to high levels of excess cholesterol in the ER. Neurotoxic agents and oxidative stress enhance ACAT1 activity which is more expressed in neurons than in glial cells (Bryleva et al., 2010; Karten, Campenot, Vance, & Vance, 2006).

In the “active” demyelinating white matter of ALD brain, there is a marked excess of cholesterol esters containing VLCFA and a diminished amount of free cholesterol when compared to control white matter or “intact” ALD white matter (Igarashi, Schaumburg, et al., 1976a; Theda, 1988; Theda et al., 1992; Table 2 from Theda et al., 1992 used with permission). The normal level of cholesterol esters and abnormal VLCFA content in the “intact” ALD white matter indicate that the accumulation of VLCFA containing cholesterol esters in the “active” demyelinating white matter is a secondary phenomenon. The excess VLCFA in macrophages and microglia scavenged from myelin debris cannot be degraded due to the lack of ALDP. The increased ACAT1 activity in response to inflammation and oxidative stress leads to increased cholesterol esters with VLCFA (Igarashi, Schaumburg, et al., 1976a; Ramsey & Davison, 1974; Reinicke, Knoll, Pretorius, Steyn, & Simpson, 1985; Yao & Dyck, 1981). The concentration of brain cholesterol esters is usually maintained at a low level as cholesterol hydrolases can convert the esters back to unesterified cholesterol; however, there is very low activity of the cholesterol ester hydrolases toward cholesterol esters with VLCFA (Ogino & Suzuki, 1981).

#### 4.2.2 Cholesterol metabolism in ALD adrenal cortex and Leydig cells

There is a marked excess of cholesterol esters containing VLCFA in ALD adrenal cortex and Leydig cells (See Section 6.3).

#### 4.2.3 Oxidized cholesterol species in ALD

Oxidized cholesterol species in ALD may play a role in inflammation (See Section 5.5).

#### 4.2.4 Cholesterol transport in ALD

Excess cholesterol can also be transported out of the brain after converting to either 27‐hydroxy cholesterol, 27‐HC, by the mitochondrial enzyme CYP27A1 or to 24‐hydroxy cholesterol, 24‐HC, by the plasma membrane enzyme CYP46A1. 27‐HC and 24‐HC are the forms of cholesterol that can cross the blood‐brain barrier, BBB. Increased permeability of the BBB to sterol molecules is related to BBB impairment (Saeed et al., 2014). 24‐HC is 30–1500‐fold higher in the brain than any other organ except the adrenal. Of interest to our understanding of the ages when there is greatest risk of development of childhood cerebral ALD is the fact that in human plasma the ratio of 24‐HC to cholesterol is 5 times higher in the first decade of life than the sixth (Lütjohann et al., 1996; Dietschy and Turley, 2004; Saeed et al., 2014). The mitochondria of non‐nerve cells, including the astrocytes, microglia and macrophages, have the enzyme CYP46A1 which can be upregulated by reactive oxygen species, ROS, in response to stress. The 24‐HC after crossing the BBB can activate Liver X receptor, LXR (Anchisi, Dessi, Pani, & Mandas, 2012; Petrov et al., 2016).

In 2015, the Bao‐Liang Song research group demonstrated that cholesterol transport is abnormal in ALD fibroblasts and in the Abcd1 mouse model for ALD as well as in other peroxisomal disorders. Low‐density lipoprotein (LDL)—derived cholesterol was transported from the lysosome to the peroxisome in a manner that depended upon lysosomal synaptotagmin VII binding to the peroxisomal lipid phosphatidylinositol 4, 5‐bisphosphate [PI(4,5)P_2_] on the peroxisomal membrane (Chu et al., 2015; Hu et al., 2018; Islinger, Voelkl, Fahimi, & Schrader, 2018; Jin, Strunk, & Weisman, 2015; Luo, Jiang, Yang, & Song, 2018; Luo, Liao, Xiao, & Song, 2017; Stefan et al., 2017). While these findings were initially called into question by van Veldhoven et al. in a letter to the editor (van Veldhoven, Baes, & Fransen, 2015), correctly identifying an error in Chu et al.'s method, a recent follow‐up publication (Xiao et al., 2019) validates the initial Chu et al. (2015) findings.

As cholesterol is an important component of many cellular membranes and is also the substrate for the synthesis of bile acids, steroid hormones and regulating oxysterols, disruption of cholesterol transport may have widespread consequences in cholesterol homeostasis (Diotel et al., 2018; McDonald & Russell, 2010; McMillan & DeMorrow, 2016).

## 5 OXIDATIVE AND ENDOPLASMIC STRESS AND INFLAMMATION IN ALD

Even in the early pathological reports of the cerebral form of ALD, the role of inflammatory changes in cerebral tissues was discussed (Powers, Liu, Moser, & Moser, 1992). With the advanced understanding of oxidative and inflammatory processes over the past decades, these have been explored in more detail in ALD.

### 5.1 Longer fatty acid chains directly induce apoptosis via endoplasmic reticulum, ER, stress response pathways

The unfolded protein response, UPR, is a cell mechanism to maintain homeostasis which can induce apoptosis when the cell is under stress. UPR is distinguished by the action of three ER‐located transmembrane receptors (protein kinase RNA‐like endoplasmic reticulum kinase [PERK], activating transcription factor [ATF6] and inositol requiring kinase [IRE1]), which regulate these events in concert. VLCFA induced ER stress in human ALD fibroblasts, correlates with the FA chain length (van de Beek et al., 2017). Equimolar concentrations of increasing length FA, of methylated C16:0, C18:0 and C20:0 do not induce ER stress, but the methyl esters of the VLCFA, C22:0, C24:0 and C26:0 do so in an increasing manner with C26:0 inducing the highest ER stress marker XB1_s_ mRNA, an active transcription factor for UPR (Maly & Papa, 2014). Consistent with VLCFA induced ER stress seen in cell culture, the PERK pathway is shown to be activated in the spinal cord of *ABCD1* knockout mice and brain and fibroblast samples from ALD patients (van de Beek et al., 2017). Both antioxidant and tauroursodeoxycholic, TUDCA, bile acid treatments of X‐ALD mice prevent ER stress activation and halt subsequent axonal neurodegeneration (Launay et al., 2017; Tabak, Braakman, & Zand, 2013). Both the antioxidant and TUDCA bile acid therapies are FDA approved and offer some hope for treatment of the UPR in AMN patients (Launay et al., 2017).

### 5.2 Fatty acids modulate mitochondrial function and increase radical generation

Mitochondria and peroxisomes are critical organelles for ROS generation. Control and *ABCD1*‐astrocytes do not show different energy‐dependent parameters (ROS generation, mitochondrial membrane potential (MMP), ADP‐dependent respiration), suggesting that *ABCD1* has no direct effect on functional mitochondrial parameters (Kruska, Schonfeld, Pujol, & Reiser, 2015). Exposure to VLCFAs C22:0, C24:0 and C26:0 increases ROS generation in both control and *ABCD1* knock out astrocytes (Kruska et al., 2015).

There are multiple identified mechanisms of lipid induced modulation of mitochondrial function and related downstream effects. First, C22:0 is shown to modulate the internal mitochondrial membrane (IMM) by inducing mild uncoupling causing an increase in resting respiration. Second, fatty acids impair electron transport and inhibit F0F1‐ATP synthase plus adenine nucleotide translocase, impairing respiration (Kruska et al., 2015). Finally, the FA‐induced impairment of electron transport is well established and hypothesized to be due to membrane destabilization by the incorporation of VLCFAs, and/or by dissociation of cytochrome c from the IMM (Di Paola, Cocco, & Lorusso, 2000; Korge, Honda, & Weiss, 2003; Reiser, Schonfeld, & Kahlert, 2006). FA‐induced impairment of calcium homeostasis is chain length dependent, seen by C20:0 and not C16:0, inducing ROS generation leading to rapid cell death of rat astrocytes (Hein, Schonfeld, Kahlert, & Reiser, 2008). C22:0, C24:0 and C26:0 have been shown to greatly increase calcium in rat glial cells, with C22:0 and partially C24:0 proving detrimental to the inner mitochondrial membrane and inhibiting phosphorylating respiration.

### 5.3 VLCFA‐induced radical generation leads to cell death

Oxidative stress is understood as an imbalance between the production of reactive oxygen species and antioxidant systems. The brain provides relatively low levels of antioxidant defense with high contents of lipid moieties such as polyunsaturated fatty acids (PUFA) and catecholamines which are especially susceptible to free radicals (Halliwell & Gutteridge, 2015). C26:0 is shown to induce DNA damage in C6 rat glial cells, with rescue effects seen via antioxidant co‐culture (Marchetti et al., 2018).

While rigorous studies have underlined the role of free fatty acids in pathology, levels of C26:0 or similar VLCFA have not been shown to correlate to disease severity, differentiate or predict phenotype. However, plasma and cellular antioxidant capacity and ROS generation have been retrospectively associated to disease phenotype (Kemp et al., 2012).

### 5.4 Antioxidant levels and the triglyceride metabolism are protective and high in AMN, not cerebral ALD

An Increase in free radical levels and a lowered antioxidant capacity in human ALD blood plasma have been shown, with cerebral patients showing depleted levels of total glutathione (Turk et al., 2017; Vargas et al., 2004). This reduced antioxidant defense is also seen in AMN fibroblast and erythrocytes (Vargas et al., 2004), with higher amounts of DNA damage induced by increased radicals in AMN leukocytes (Marchetti et al., 2015).

Distinguishing markers between AMN and cerebral ALD is of critical importance, in both understanding the pathology and in creating a predictive biomarker for the cerebral form of ALD. Lipid and transcriptomics performed in human ALD fibroblasts revealed the differences between AMN and childhood CALD metabolism, suggesting that an increased triglyceride metabolism plays a protective role in AMN which is absent in cerebral disease (Lee et al., 2019). The pathways decreased in cerebral ALD were related to typical lipids and not VLCFAs (Lee et al., 2019). In AMN, anabolism in sphingolipid pathways including sphingomyelin and glycosphingolipid were downregulated and catabolism up‐regulated. In AMN, the upregulated triacylglycerol metabolism is interesting, as triglycerides have shown neuroprotective effects against fatty acid‐induced lipotoxicity (Listenberger et al., 2003) by keeping some excess saturated or monounsaturated fatty acids in lipid droplets and preventing the induction of necroptosis in oligodendrocytes and astrocytes (Hein et al., 2008; Parisi, Li, & Atilla‐Gokcumen, 2017). Triglycerides containing PUFAs limit toxicity by preventing PUFA‐phospholipids from undergoing oxidation and increasing oxidative stress (Jarc et al., 2018; Li, Sancak, Frasor, & Atilla‐Gokcumen, 2018). One repeated question is whether these protective mechanisms are absent in cerebral patients before the onset of disease, and may serve as a predictive biomarker, or change as a result of or as a cause of cerebral disease onset. The importance of robust natural history studies in the AMN population including tissue sampling is highlighted by these findings.

Early oxidative damage to proteins of the spinal cord in the *Abcd1^−^* mouse was detected at 3.5 months of age, well before onset of symptoms. Bovine Serum Albumin, BSA‐C26:0 fatty acid was added to cultured fibroblasts from ALD and controls and showed increase of reactive oxygen species, ROS, decreased levels of glutathione and diminished mitochondrial membrane potential in ALD cells but not in controls. Cells cultured in the presence of the lipid antioxidant, α‐tocopherol, showed a reduction in oxidative damage. This experimental data led to the antioxidant trial in AMN patients (Fourcade et al., 2008).

### 5.5 Oxidized cholesterol species may contribute to inflammation

Oxidative stress causes the oxidation of cholesterol leading to the formation of cholesterol oxide derivatives oxidized at C7; 7‐ketocholesterol (7KC), 7β‐hydroxycholesterol and 7α‐hydroxycholesterol. 7KC was found to be increased in plasma from ALD patients. Addition of 7KC to the culture media of BV‐2 cells, the murine model of glial cells, induces changes in peroxisomal functions. 7KC induces overproduction of H_2_O_2_ and $O_{2}^{-}$ and several peroxisomal modifications: decreased Abcd1, Abcd2, Abcd3, Acox1 and Mfp2 mRNA and protein levels, increased catalase activity and decreased Acox1 activity. These findings suggest that high levels of 7KC in ALD plasma could intensify brain damage (Nury et al., 2017).

The synthesis of 25‐hydroxycholesterol, 25‐HC, is catalyzed by the enzyme cholesterol 25‐hdroxylase, CH25H, which uses cholesterol and molecular oxygen as substrates and NADPH as a cofactor. 25‐HC has potent and wide‐ranging effects in the immune system including the differentiation of monocytes to macrophages (McDonald & Russell, 2010).

Increased expression of CH25H was found in primary fibroblasts in one out of three AMN and in all three CALD subjects when compared with controls. The levels of 25‐HC were increased in all CALD cell lines. The authors also generated oligodendrocyte precursor cells, OPCs, from controls, AMN and CALD subjects. Consistent with the findings in primary cells, CH25H mRNA levels were significantly higher in the CALD‐OPCs than control and AMN OPCs (Jang et al., 2016). Exogenous addition of 1 µM 25‐HC to CALD fibroblasts and OPCs led to a reduction in the C26/22 ratio. The 25‐HC was found to downregulate ELOVL1 as well as activating LXR (Jang, Lee, Song, Kim, & Min, 2019).

## 6 CELL‐ AND TISSUE‐SPECIFIC PATHOLOGY

### 6.1 Pathology and biochemical changes in the ALD brain

Males and females with ALD are born with normal brain function. Myelination occurs normally and there is no developmental delay (Berger, Forss‐Petter, & Eichler, 2014). However, about 60% of ALD males develop a rapidly fatal demyelinating disease, with about 35% occurring in childhood between 3 and 10 years, about 5% in adolescence, before the onset of spinal cord disease and another 20% as adults after years of spinal cord disease.

In normal appearing white matter of postmortem brains of ALD cases, a 17‐fold excess of C26:0 was found in phosphatidylcholine compared with white matter of controls (Theda et al., 1992). Sharp, Johnson, and Poulos (1991) also found an excess of C30:0, C32:0 and C34:0 in phosphatidylcholine. The other phospholipids, cholesterol esters, triglycerides and the sphingolipids either had normal levels, or less than a twofold elevation (Theda et al., 1992). The presence of saturated VLCFA in phosphatidylcholine in myelin may lead to instability and inflammation. The VLCFA desorb much slower than normal dietary fatty acids, FA, from both albumin and protein‐free lipid bilayers. As VLCFA accumulate due to impaired peroxisomal β‐oxidation and enhanced FA elongation, elevated levels of VLCFA in membranes could alter structure and function in myelin (Ho et al., 1995).

The initiation of demyelination is still not well understood. There is no phenotype/genotype correlation as all forms of ALD occur with the same mutation in the *ABCD1* gene, within the same kindred and even differing time of onset in monozygotic twins (Di Rocco, Doria‐Lamba, & Caruso, 2001; Korenke et al., 1996). The levels of VLCFA in cultured cells and blood are the same when comparing AMN and cerebral ALD, CALD. CALD is rare in ALD females and the few reported cases are due to a Xq27‐ter deletion of the non‐mutated X, or skewed X‐inactivation favoring the mutated X (Hershkovitz et al., 2002; Maier et al., 2002; Migeon et al., 1981). However, the amounts of VLCFA levels in normal appearing white matter were found to be higher in CALD compared with the levels in pure AMN (Asheuer et al., 2005). The study of oligodendrocytes derived from pluripotent stem cells from CALD accumulated more VLCFA than those derived from AMN patients (Jang et al., 2019). The blood–brain barrier may be leaky secondary to oxidative stress caused by increased levels of saturated VLCFA in myelin and cellular membranes (Lauer et al., 2017). In vitro studies suggest lack of *ABCD1* causes endothelial dysfunction preceding the accumulation of VLCFA (Kemp et al., 2016; Musolino et al., 2015). Early presence of contrast enhancement on magnetic resonance imaging, MRI, after brain contusion suggest that the disruption of the blood–brain barrier maybe the trigger for inflammatory demyelination (Aubourg, 2015; Raymond et al., 2010). Thus, monocytes and activated macrophages can enter the brain to scavenge cellular and myelin debris. ALD patients have normal capacity for macrophage differentiation and phagocytosis; however, they are pro‐inflammatory skewed in both CALD and AMN (Aubourg, 2015; Musolino et al., 2015; Musolino, Rapalino, Caruso, Caviness, & Eichler, 2012). *ABCD1* deficiency leads to incomplete establishment of anti‐inflammatory responses of macrophages possibly contributing to the rapidly progressive demyelination in CALD (Weinhofer et al., 2018).

The demyelination begins usually in the splenium of corpus callosum where the white matter fiber bundles are most tightly packed and spreads outward into the periventricular white matter (Powers et al., 1992; Schaumburg, Powers, Raine, Suzuki, & Richardson, 1975; Schaumburg, Powers, Suzuki, & Raine, 1974). This is the area where the oligodendrocytes express the highest levels of ABCD1 (Lauer et al., 2017).

Interestingly, the corpus callosum white matter microstructure has more recently been shown to be the most sensitive region to repetitive head trauma in competitive sports players, who were clinically unremarkable and cognitively unaffected. Changes in diffusion tensor imaging metrics were seen after one season of college‐level American football (Koerte et al., 2015; McAllister, 2014) and metabolic reductions in the N‐acetylaspartate/creatine‐phosphocreatine ratio (NAA/Cr) in the corpus callosum, measured by single‐voxel MR spectroscopy were seen in subclinical hockey players. These changes indicate a relationship between head impact exposure and white matter microstructural changes. As traumatic brain injury has been shown to induce cerebral disease in ALD, one may speculate that biomechanically induced torsion stresses upon the intra‐hemispheric callosal connection under regular physical activity levels may predispose the location as more susceptible to initial blood brain barrier breakdown by causing microvascular endothelial damage constituting a possible longitudinal “hit” in the multiple hit model of cerebral disease onset (Singh & Pujol, 2010).

There are marked changes in the lipid profile of ALD brain tissue when active demyelinating areas are analyzed in postmortem tissue. The VLCFA are enriched in cholesterol esters, predominately located in invading monocytes/macrophages entering the brain following the opening of the blood–brain barrier (Schaumburg et al., 1974). The myelin lipids, the phospholipids, sphingomyelin, cerebrosides, sulfatides, gangliosides and the proteolipid protein show an increased VLCFA (Bizzozero, Zuniga, & Lees, 1991; Kishimoto et al., 1984; Theda et al., 1992). The anti‐inflammatory plasmenylethanolamines (PlsEtn) are markedly decreased and there are increased levels of reactive lipid aldehydes and oxidized proteins leading to elevations of reactive oxygen species, ROS (Khan et al., 2008; Theda et al., 1992). The reduction of PlsEtn in CALD brain may be due to the increased need of the anti‐inflammatory polyunsaturated fatty acids stored in the sn2 position of PlsEtn and released by phospholipase A2, and the decreased peroxisomal synthesis of PlsEtn, in part, by the lack of acetyl CoA generated by peroxisomal β‐oxidation (Farooqui, Ong, & Horrocks, 2003; Hayashi & Oohashi, 1995).

The three‐hit hypothesis describing the pathomechanisms of CALD is: (a) the metabolic defect including an increase in VLCFA leading to axonal degeneration and oxidative stress. (b) Neuroinflammatory processes, which may stem from environmental, stochastic, genetic or epigenetic factors resulting in macrophage infiltration and production of higher levels of inflammatory mediators. (c) After macrophage production of pro‐inflammatory cytokines and chemokine mediators cause loss of peroxisomal functions including VLCFA oxidation and this leads to greater accumulation of VLCFA (Singh & Pujol, 2010). Here, the macrophages and microglia which have taken up the myelin debris cannot degrade the overabundant VLCFA and thus the cascade of metabolic stress and loss of myelin continues.

### 6.2 Pathology and biochemical changes in spinal cord of AMN patients

Most men with ALD will develop slowly progressive myeloneuropathy in their 20s or 30s. About 65% of ALD women will also develop symptoms of spinal cord disease by the age of 65, although some women may have symptoms in their 20s (Engelen et al., 2014). Early symptoms of AMN are loss of sensation in the legs followed by the development of a spastic gait and bladder and bowel incontinence. The peripheral nerves are also involved, and in ALD women there is often dysesthesia (van Geel et al., 1996). The increased VLCFA in the myelin lipids of the AMN spinal cord cause oxidative stress and impaired mitochondrial function that contribute to the myeloneuropathy through a failure of ATP‐dependent axonal transport (Fourcade et al., 2008; Wanders, 2014). This leads to a distal dying‐back axonopathy. The peripheral nerves are also involved, with primary axonal degeneration in most AMN men and 80% of women. Histological analyses of the dorsal root ganglia from AMN spinal cord did not show apparent neuronal loss, necrosis or apoptosis, a non‐inflammatory myopathy (Powers, DeCiero, Ito, Moser, & Moser, 2000). On ultrastructural analysis, many neurons contain mitochondria with lipid inclusions leading to the failure of ATP‐dependent axonal transport in AMN spinal tracts. There was loss of large axons that were replaced with smaller axons. Impaired mitochondrial function may contribute to the dying back axonal degeneration (Powers et al., 2001). Recently, Gong et al. described the expression of “eat‐me” molecules MFGE8 and TREM2 preceding complement activation and synapse loss in the spinal cord. C26:0‐LPC added to ABCD1‐deficient microglia in culture induced MFGE8 expression, aggravating phagocytosis leading to neuronal injury (Gong et al., 2017).

### 6.3 Pathology and biochemical changes in ALD adrenal cortex and Leydig cells

The lifetime incidence of adrenal disease in ALD males is about 80% with 46.7% developing adrenal hormone deficiency in childhood, 6 months–10 years of age, 28.6% ages 10–40 years and only 5.6% after the age of 40 years (Huffnagel, Laheji, et al., 2019b). The fetal adrenal in ALD males has the abnormal pathology and biochemistry found in adrenals of postnatal males with ALD. In the adrenal cortex, there is striking accumulation of cholesterol esters with VLCFA. These are increased to 30% in ALD versus 1%–3% in controls (Igarashi, Schaumburg, et al., 1976a; Moser et al., 1982). Cholesterol esters containing VLCFA are very poor substrates for the cholesterol ester hydrolases (Ogino & Suzuki, 1981). This leads to their accumulation within the cell in the form of lamellar inclusions and to cell dysfunction and cell death (Powers, Schaumburg, Johnson, & Raine, 1980). When C26:0 and C24:0 fatty acids were added to the culture media of primary adrenal cortical cells in a 5 μM concentration there was increased membrane microviscosity of the cells and a reduction to adrenocorticotropic hormone, ACTH, stimulation (Whitcomb, Linehan, & Knazek, 1988). This study suggests that there may be direct toxicity of the VLCFA. There is also evidence that due to the accumulation of cholesterol esters with VLCFA, there may not be enough free cholesterol available for synthesis of the steroid hormones (Powers, 1985). VLCFA accumulate in the zona reticularis and zona fasciculate with sparing of the zona glomerulosa leading to primary cortisol insufficiency and androgen deficiency. The site of adrenal dysfunction is in good agreement with the fact that ABCD1 protein is found in the adrenal cortex but not in the adrenal medulla, while ABCD2 shows the opposite distribution (Troffer‐Charlier et al., 1998). The VLCFA in the cell membranes may also interfere with ACTH and gonadotropin binding to their receptors (Burtman & Regelmann, 2016; Huffnagel, Laheji, et al., 2019b). Similar biochemical changes and pathology are found in the Leydig cells of the testes leading to defective hormonogenesis. Thus, men with ALD may have clinical and subclinical hypogonadism and impaired sexual function although many have fathered children. Low levels of testosterone with elevation of luteinizing hormone, LH, and follicle‐stimulating hormone, FSH, concentrations are consistent with defective testicular function (Powers & Schaumburg, 1981). Adrenal function should be monitored in all ALD males and can be corrected with administration of adrenal hormones, with stress dosing during illness, accident or surgery. Adrenal insufficiency is rare in females with ALD (Dubey et al., 2005; Engelen et al., 2014; Huffnagel, Laheji, et al., 2019b).

### 6.4 Searches for modifier genes

There is considerable interest in finding genes that modify the phenotype of ALD. A polymorphism in CYP4F2, the gene that is responsible for peroxisomal ω‐oxidation of VLCFA to very long‐chain dicarboxylic acids was found to increase the risk of development of childhood onset CALD by decreasing the clearance of VLCFA through ω‐oxidation (van Engen et al., 2016). Eleven microRNAs were identified that had different expression for CALD and AMN and one of them, MiR‐196a, was found to inhibit the expression of two inflammatory signaling factors as well as ELOVL1 (Shah & Singh, 2017).

## 7 EXPERIMENTAL MODELS

### 7.1 Animal models

Animal models provide a necessary platform in understanding the pathology of disease and serving as a test bed in demonstrating the biological significance of novel therapeutic interventions. In commonly used genetically malleable species such as the drosophilae, mouse and zebra fish, the equivalent *ABCD1* gene has been successfully targeted to generate knock‐out models, each providing a unique strength and limitation (Forss‐Petter et al., 1997; Gordon, Valdez, & Letsou, 2018; Kobayashi, Shinnoh, Kondo, & Yamada, 1997; Lu et al., 1997; Strachan et al., 2017).

#### 7.1.1 Drosophilae models

In drosophilae models, *CG2316* has been identified as a sole homolog of human *ABCD1*, termed *dABCD*. Transgenic *dABCD* transgenic flies survive to adulthood with a neurodegenerative phenotype neuron and glia loss. A same phenotype is seen in long‐/very long‐chain acyl‐CoA synthetase gene *bgm* models. Disruption of *dABCD* in neurons results in retinal defects, which are not caused by the same alterations to glial cells. In the drosophilae model, environmental stress modifies penetrance and expressivity of neurodegeneration (Gordon et al., 2018). Interestingly, here the phenotype is rescued (reduction of retinal damage) with diet supplementation by medium‐chain‐FA; however, supplementation by long‐chain FA does not exacerbate disease.

#### 7.1.2 Zebra fish model

A zebra fish model (*Danio Rerio*) with *ABCD1* mutants shows elevated VLCFA levels, with hypomyelination of the spinal cord, abnormal patterning and decreased numbers of oligodendrocytes and increased cell death. Functionally, the fish demonstrate impaired motor function and a decrease in overall survival. Induction of human ABCD1 expression in oligodendrocytes reduced embryonic apoptosis of these cells and improved motor function (Strachan et al., 2017).

#### 7.1.3 Murine models

In 1997, three mouse models for ALD, were developed separately by researchers in, Vienna, Austria, Fukuoka, Japan and Baltimore, USA (Forss‐Petter et al., 1997; Kobayashi et al., 1997; Lu et al., 1997).

The *Abcd1* deficient mouse is a well investigated yet challenging model. While biochemical defects have been measured early in life, with oxidative biomarkers present at as early as 3.5 months, the mouse does not develop the deadly cerebral phenotype seen in humans. As the *Abcd1‐*deficient mouse most resembles the AMN phenotype, developing axonopathy and locomotor impairment at a very late 22 months of age (Pujol et al., 2002); it has been the model of choice for novel therapeutics targeting the spinal cord. A double‐mutant *Abcd1*/*Abcd2* mouse exhibits higher VLCFA accumulation in the spinal cord (Pujol et al., 2004), higher levels of oxidative damage (Fourcade et al., 2010) and more severe locomotor impairment with earlier onset (Fourcade et al., 2008). In this mouse, axonopathy progression was halted and biochemical markers of oxidation and ER stress were ameliorated by antioxidant compound and TUCDA bile acid therapies. (Fourcade, Lopez‐Erauskin, Ruiz, Ferrer, & Pujol, 2014; Launay et al., 2017; Lopez‐Erauskin et al., 2011) In the peripheral nerves of the *Abcd1* mouse, Kleinecke demonstrated lysosomal storage and increased VLCFA in gangliosides of lipid rafts causing altered axonal Kv1 channels in Schwann cells leading to peripheral neuropathy which was age dependent (Islinger et al., 2018; Kleinecke et al., 2017).

Double‐knockout mouse models were developed with other PEX genes, the *Pex7/Abcd1*, and the *Abcd1* with the peroxisome‐deficient oligodendrocytes that do have some pathology resembling that in patients with ALD (Kassmann et al., 2007). The *Pex7/Abcd1* model shows demyelination in white matter suggesting that plasmalogens may have both structural and functional roles in membrane and cellular stability (Brites, Mooyer, Mrabet, Waterham, & Wanders, 2009).

As ELOVL1 is the essential enzyme in chain elongation of C22:0 to C26:0. Transgenic mice overexpressing ELOVL1 in oligodendrocytes (*Cnp‐ELOVL1* ^±^) had a 25‐fold higher elongation activity for C22:0‐CoA. Double transgenic *ABCD1^y/^* ^−^, *Cnp‐ELOVL1* ^±^ mice had C26:0 levels increased 20‐fold in brain, 44‐fold in adrenals, 90‐fold in testes and increased in other organs more than what was found for the single knockout of *ABCD1^y/^* ^−^. One of the interesting conclusions of this study was that oligodendrocytes are largely responsible for total VLCFA synthesis in the brain. In addition, the studies showed that the levels of C24:0‐lysoPC, C26:0‐lysoPC and C22:0‐carnitine, C24:0‐carnitine and C26:0‐carnitine were increased in brain and spinal cord from *ABCD1^y/^* ^−^ and *ABCD1^y/^* ^−^, *Cnp‐ELOVL1^±^* mice (van de Beek et al., 2016).

### 7.2 Cellular model

Recently a microglial model for ALD was produced by knocking out *Abcd1 and Abcd2* in BV‐2 cells, murine microglial cells. These cells accumulate VLCFA and have lipid droplets and striated and whorled lipid inclusions (Raas et al., 2019).

## 8 DIAGNOSTIC TESTING FOR ALD AND THE DEVELOPMENT OF NEONATAL SCREENING

### 8.1 Diagnostic testing for ALD

Historically, the diagnosis of ALD in boys was established by neuroimaging following early symptoms of attention deficit and hyperactivity disorder, failure in school, difficulties in understanding language, behavior disturbances and decline in handwriting (Moser et al., 2001). In the early 1980s, the measurement of very long‐chain fatty acids in plasma provided a reliable diagnostic test (Moser et al., 1981). It allowed that the diagnosis suspected from neuroimaging (initially computer tomography and later MRI) to be confirmed biochemically by measurement of plasma total lipid VLCFA (Moser et al., 1999). Once the *ABCD1* gene was known, the sequencing of the *ABCD1* gene was used diagnostically as analysis of the family *ABCD1* mutation improves the diagnosis of ALD women as the plasma VLCFA test has a 20% false negative rate (Boehm, Cutting, Lachtermacher, Moser, & Chong, 1999).

Following the development of the whole blood spot liquid chromatography tandem mass spectroscopy (LC‐MS/MS) measurement of C26:0‐lysophosphatidyl choline, C26:0‐LPC, for the diagnosis of newborns with ALD, the Academic Medical Center in Amsterdam has shown that the C26:0‐LPC assay accurately diagnoses males with ALD, and importantly offers an accurate diagnostic test for women with ALD, with a reported sensitivity of 100% in 49 females and 126 controls (Huffnagel et al., 2017).

In support of this finding, we present previously unpublished C26:0‐LPC measurements by LC‐MS/MS in 1/8″ dry blood spot, DBS, in189 controls and 117 ALD heterozygote females. Setting the diagnostic threshold at the maximum healthy control measurement (0.1578 μMol) for a specificity of 100, C26:0‐LPC demonstrates a sensitivity of 94.87% (95% CI = 89.17%–98.1%) in differentiating between healthy control and women with ALD, with an overlap seen in six measurements in the female group (Supporting Information Method S1 and Figure S1).

### 8.2 The development of newborn screening for ALD

**Figure 1:**
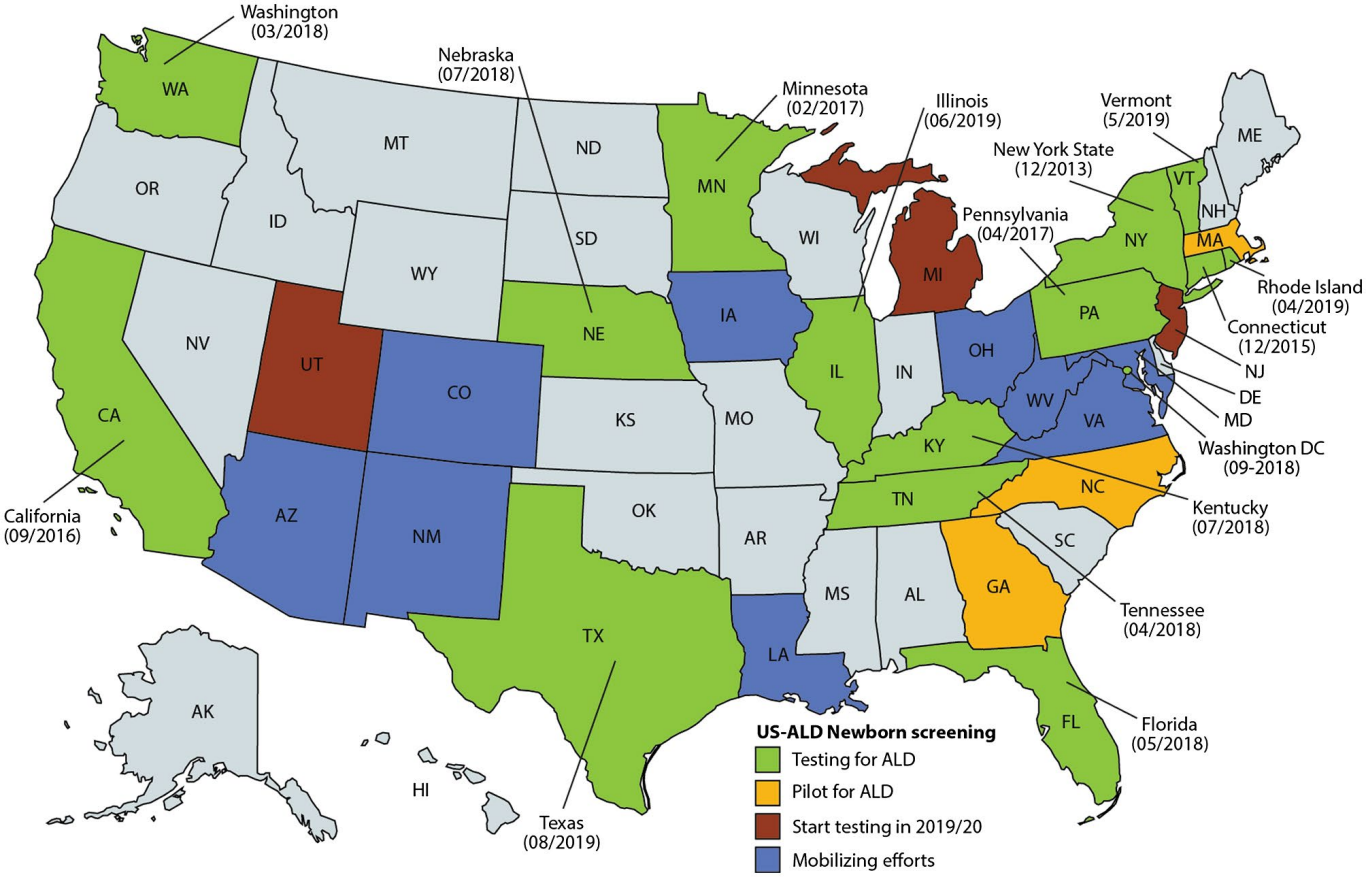
ALD newborn screening in the USA as of October 19, 2019 (https://adrenoleukodystrophy.info)

Hematopoietic stem cell transplantation, HSCT is live‐saving gold standard, initiated as soon as cerebral disease is discovered. However, as neurological deficits do not improve with therapy, improving diagnosis would allow for surveillance of cerebral disease and reduce potential residue (Mahmood, Raymond, Dubey, Peters, & Moser, 2007; Moser et al., 2000). Thus, neonatal screening was proposed for ALD by Hugo Moser and colleagues in 2004. However, at that time there was no valid test for ALD using the newborn blood spot. Informed by analyses of lipids in normal appearing white matter of ALD brain, which showed that C26:0 was increased in phosphatidylcholine (Theda, 1988; Theda et al., 1992), the potential to diagnose ALD in blood phospholipid subfractions was investigated. In 2006 a first newborn screening test for ALD by measurement of C26:0‐lysophosphatidylcholine, C26:0‐LPC in newborn dried blood spot was established (Haynes & De Jesus, 2012; Hubbard et al., 2009, 2006; Raymond, Jones, & Moser, 2007; Theda et al., 2014; Turgeon et al., 2015). After refining the LC‐MS/MS assay of C26:0‐LPC and several pilot studies, in December 2013, neonatal screening for ALD started in the state of New York. In February 2016, the Secretary of Human Health signed the recommendation to add ALD to the recommended uniform screening panel in the USA (Kemper et al., 2017; Moser et al., 2016). As of October 19, 2019, 14 states and the District of Columbia are screening all newborns for ALD, 3 states have ALD pilot screening, and more states are planning to start within the next year (Figure 1). In October 2019, the Netherlands plans to start an ALD pilot screening of all newborn males. The early detection of the biochemical abnormalities associated with ALD and AMN has proven to be reliable to detect those affected by the condition but also poses new ethical and clinical challenges (Kemper et al., 2017; Moser et al., 2016).

## 9 ASSESSMENTS OF DISEASE SEVERITY

With the advent of newborn screening (or when relatives are diagnosed), early confirmation of the biochemical and genetic abnormalities associated with the diagnosis of ALD/AMN is possible. While the early, presymptomatic diagnosis of ALD/AMN might be quite distressing to the families of those affected, it is an important step to minimize morbidity and mortality. To be able to provide best evidence‐based care for children and their families, especially with a focus on preventing the devastating advanced forms of CALD, the establishment of reliable imaging criteria and biomarkers that should trigger interventions is essential.

### 9.1 Adrenal function

Adrenal dysfunction should be closely monitored in all males (Regelmann et al., 2018). Adrenal hormone therapy is successful at preventing severe illness or loss of life due to Addisonian crisis (Burtman & Regelmann, 2016; Huffnagel, Laheji, et al., 2019b; Shulman, Palmert, Kemp, Lawson Wilkins, & Therapeutics, 2007).

### 9.2 Neuroimaging

MRI with or without contrast enhancement is used to monitor presymptomatic males with ALD for early and progressive white matter changes (Liberato et al., 2019; Eichler et al., 2007; Loes et al., 2003). A semi‐quantitative MRI severity score was developed by Daniel Loes, referred to as the Loes score, with 0.5 or less for “normal” ranging to 34 at maximum severity. For those known to be affected by ALD (after newborn screening or through screening due to affected relatives), close monitoring by neuroimaging is recommended.

### 9.3 Other assessments

Employed in the investigative setting, assessment of physical capabilities and measurements of walking speed, hip strength, vibration sense and nerve conductions studies has been used in assessing disease severity in AMN (Zackowski et al., 2006). A 2‐year study of disease progression in males with AMN used Expanded Disability Status Score (EDSS), a Severity Scoring System for Progressive Myopathy (SSPROM), quantitative vibration measurement at hallux, the 6‐min walk test and timed up‐and‐go to assess the progression of myelopathy (Huffnagel, Ballegoij, et al., 2019c).

## 10 STANDARD THERAPY FOR CHILDHOOD CEREBRAL ALD

Currently, effective treatment for early brain disease, detected by MRI with contrast enhancement, is allogenic hematopoietic stem cell transplantation, HSCT. Ideal candidates for intervention are individuals with a Loes score of 9 or lower, without any neurologic deficits, who receive HLA‐matched sibling or related donor HSCT. However, this intervention has a high‐morbidity and long‐term sequelae related to immunosuppression and graft versus host disease. It is important to note that disease progression continues for some 6–9 months following HSCT (Miller et al., 2011; Peters et al., 2004; Raymond et al., 2019). Importantly, adrenal dysfunction is not corrected following HSCT transplant for cerebral disease (Burtman & Regelmann, 2016).

## 11 EXPERIMENTAL THERAPEUTIC TRIALS AND STRATEGIES

### 11.1 Dietary therapies; Lorenzo's oil

As the VLCFAs play a crucial role in the pathogenesis of ALD, the first trials to normalize the VLCFA in ALD through dietary restriction of VLCFA, followed by oleic acid alone and then Lorenzo's oil, a 4:1 mixture of oleic and erucic acid triglycerides, were tried, but all were without neurological or endocrine improvement (Moser et al., 2001). Lorenzo's oil together with a low‐fat diet was given to 89 asymptomatic ALD boys in an open, non‐placebo‐controlled, trial. After a mean follow‐up of 6.9 years, 24% developed CALD; however, compared to historical data, the percentage of boys that would develop CALD was 37% suggesting a protective effect (Moser et al., 2005). Studies in postmortem brain of CALD who were on Lorenzo's oil showed no evidence of erucic acid in brain lipids (Poulos, Gibson, Sharp, Beckman, & Grattan‐Smith, 1994; Rasmussen, Moser, Borel, Khangoora, & Moser, 1994); however, later studies in rats showed that erucic acid entered the brain and was either degraded or chain elongated to nervonic acid (Golovko & Murphy, 2006). The mechanism for Lorenzo's oil was thought to be by competitive inhibition of the chain elongation of saturated fatty acids by providing an excess of monounsaturated fatty acid precursors. The in vitro study of Lorenzo's oil in HeLa cells expressing high levels of ELOVL1, the enzyme that catalyzes the chain elongation of C22 to C26 fatty acids in the endoplasmic reticulum, showed that Lorenzo's oil strongly inhibits ELOVL1 (Sassa, Wakashima, Ohno, & Kihara, 2014). Lorenzo's oil therapy is not FDA approved as a double‐blind placebo‐controlled trial has not seen successful completion. In open‐label studies, Lorenzo's oil has not shown to halt or slow disease progression in AMN nor in CALD (Aubourg et al., 1993; van Geel et al., 1999; Rizzo, 1993).

### 11.2 Metabolic modulators

One inhibitor of ELOVL1 is bezafibrate that showed reduced VLCFA in ALD fibroblasts (Engelen, Schackmann, et al., 2012a). However, in a clinical trial of administering bezafibrate to AMN men and women VLCFA in plasma, lymphocytes and dried whole blood spots were not lowered (Engelen, Tran, et al., 2012b).

Statins lower LDL cholesterol. In 1998 Singh et al. reported that ALD patients given lovastatin normalized their plasma VLCFA (Singh, Khan, Key, & Pai, 1998). Another randomized double‐blind crossover trial comparing lovastatin to placebo showed no normalization of C26:0 in ALD patients (Engelen et al., 2010).

Various studies have shown an upregulation of peroxisomal β‐oxidation in cells from ALD subjects and the *ABCD1* mutant mouse model by upregulation of the ABCD2 protein and peroxisome proliferation. Normalization of C24:0 levels in brain and C26:0 levels were lowered by 80% in Abcd1 mice after 6 weeks of feeding 4‐phenyl‐ butyrate, 4PBA (Kemp et al., 1998).

Overexpression of Abcd2 in an Abcd1 knockout mouse normalizes VLCFA in spinal cord, sciatic nerve and adrenal gland (Pujol et al., 2004). However, to date there have not been clinical trials of 4PBA in ALD.

It was shown that thyroid hormone receptor agonist sobetirome increased Abcd2 mRNA levels in brain and liver of wild‐type mice. Adult *Abcd1* KO mice were treated with sobetirome for 12 weeks resulting in a lowering of C26:0‐LPC levels in plasma, brain, testes and adrenal tissue by ~20% (Hartley, Kirkemo, Banerji, & Scanlan, 2017). Other thyroid hormone agonists are currently being pursued as potential therapeutic candidates in ALD.

Morato et al. demonstrated that pioglitazone, a PPARγ agonist, halts axonal degeneration in the Abcd1 mouse by restoring mitochondria (Morato et al., 2013). Recently, a derivative of pioglitazone has been developed and is currently in being assessed in a multinational, placebo‐controlled, randomized trial in AMN.

### 11.3 Anti‐inflammatory strategies

Anti‐inflammatory drugs such as immunoglobulin, cyclosporine, cyclophosphamide and interferon‐beta were tried to halt or reverse the cerebral inflammation (Moser et al., 2001). These drugs were not effective in halting the cerebral inflammation.

### 11.4 Antioxidant therapy

A combination of multiple high‐dose antioxidants was recently demonstrated to normalize biomarkers for oxidative damage and inflammation in a small open‐label trial of adult patients with AMN (Casasnovas et al., 2019). There appeared to be a potential effect on the 6‐min walk test, justifying larger placebo‐controlled trials in future.

Several centers have utilized N‐Acetyl cysteine (NAC) adjunct therapy for individuals undergoing allogeneic HSCT. One study showed individuals with more advanced MRI undergoing HSCT had improved survival with NAC therapy, not however demonstrating an improvement in residual neurologic deficit (Miller et al., 2011).

As with most neurologic disease, main pharmacodynamic challenges are in passing the blood–brain barrier and in the targeted delivery of a therapeutic compound. Recently, a nanoparticle polyamidoamine, PAMAM, dendrimer drug delivery platform conjugated to NAC (D‐NAC), having shown rescue of motor impairments and inflammatory status in a neonatal rabbit model of cerebral palsy (Kannan et al., 2012), demonstrated targeted drug delivery into *Abcd1* mouse spinal cord microglia, and in ex vivo cerebral ALD peripheral blood monocytic patient cells stimulated by VLCFA (C26:0), normalization of antioxidant and inflammatory status (Turk et al., 2018).

### 11.5 Gene therapy

In order to decrease the burden of morbidity of allogeneic HSCT, a trial of autologous HSCT with ex vivo lentiviral gene correction of CD34‐positive stem cells is ongoing(Cartier et al., 2009; Eichler et al., 2017). Interim findings of this trial in 17 ALD boys with early stage brain disease who received the Lenti‐D *ABCD1* gene therapy have been reported. No treatment death or graft versus host disease was seen. Fifteen of the boys survived; however, 1 died of rapid neurologic deterioration and the other, who had evidence of rapid disease progression on MRI, withdrew from the study to undergo allogenic stem cell transplantation and died of complications. All 15 boys who survived remained free of major functional disabilities at the 24‐month follow‐up. A longer follow‐up and larger sample size is needed to confirm the efficacy and safety of *ABCD1* gene therapy with the Lenti‐D lentiviral vector.

In addition to ex vivo lentiviral gene correction, in vivo adeno‐associated virus 9 (AAV9)‐based gene therapy is being pursued. Gong et al. have showed intrathecal delivery of an AAV9 carrying *ABCD1* in mice corrected VLCFA metabolism and behavioral outcomes (Gong et al., 2015). This therapeutic strategy may show promise for AMN due to the intrathecal delivery.

### 11.6 Other therapeutic strategies

The screening of drug libraries for drugs that reduce the VLCFA in transformed human fibroblasts is currently being investigated (Schrifl, 2016). Redirecting the synthesis of saturated VLCFA to monounsaturated VLCFA, which are less toxic to membranes, by upregulating the enzyme Stearoyl‐CoA Desaturase‐1 is another current avenue of research (van de Beek et al., 2019).

## 12 CONCLUSION

This review of ALD summarizes our understanding of *ABCD1*‐ and VLCFA‐related pathogenic mechanisms, and rationale they provide for current experimental therapeutic strategies. In ALD, the *ABCD1* mutation is shown to dysregulate manifold metabolic and immune pathways, inducing tissue‐ and cell‐specific pathogenic processes. VLCFA is shown to both directly induce apoptotic pathways, and indirectly via ER and mitochondrial radical‐related stress mechanisms. These mechanisms are shown to be chain length dependent, providing a rationale for a therapeutic approach in the reduction of specific VLCFA moieties.

Oxidative stress and antioxidant systems show phenotypic‐specific dysregulation in ALD, suggesting therapeutic benefit by antioxidant strategies. Additionally, marked reduction in other endogenous antioxidants such as the peroxisomal, myelin‐critical plasmalogen species seen in cerebral ALD brain tissue, may contribute further insight into underlying disease mechanisms. Promising results from an ongoing gene‐therapy trial and recent antioxidant therapy investigations and the increase in newborn screening programs provide hope for patients with ALD.

## CONFLICT OF INTERESTS

Ali Fatemi, MD is on the safety monitoring board for Bluebird Bio, Stealth Biotherapeutics and a paid consultant to Calico Laboratories. Christiane Theda, MD, PhD is the Co‐Founder, Chief Medical Officer and a Company Director of Navi Medical Technologies Pty Ltd and Medical Advisor to Ventora Pty Ltd. These companies develop new biomedical devices unrelated to peroxisomal disorders and adrenoleukodystrophy. Bela R. Turk, MD and Ann B. Moser, BA have no conflict of interest.
