# Supplementary figures and images for: X‐linked adrenoleukodystrophy: Pathology, pathophysiology, diagnostic testing, newborn screening and therapies
Source: Int J Dev Neurosci. 2020 Jan 26;80(1):52–72. doi: 10.1002/jdn.10003 (PMC7041623; doi:10.1002/jdn.10003)

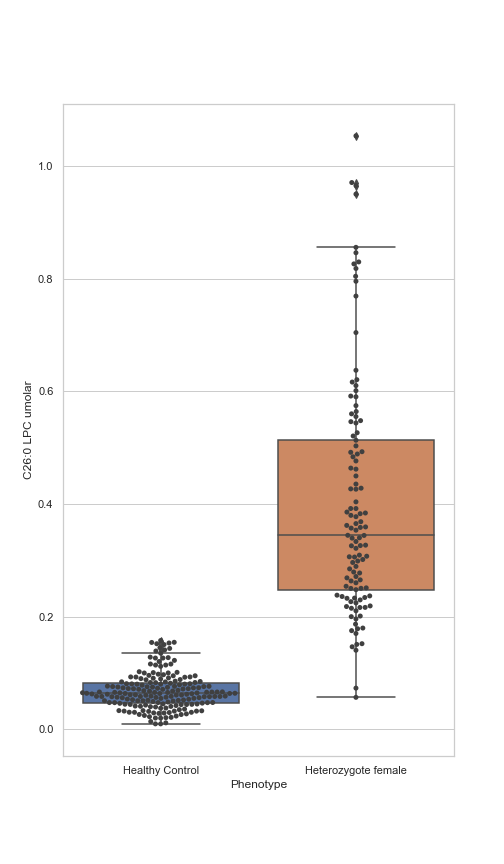

Supplement: Supplementary file 1 [file JDN-80-52-s001.tiff]
