# Supplementary material for: X‐linked adrenoleukodystrophy: Pathology, pathophysiology, diagnostic testing, newborn screening and therapies
Source: Int J Dev Neurosci. 2020 Jan 26;80(1):52–72. doi: 10.1002/jdn.10003 (PMC7041623; doi:10.1002/jdn.10003)
Supplement: Supplementary file 2 [file JDN-80-52-s002.docx]

**Figure S1:** C26:0-Lysophosphatidylcholine (C26:0-LPC) uMol in dried whole blood spots, DBS, was measured in 189 non-ALD men and women and 117 ALD heterozygotes by LC-MS/MS. Whole EDTA anti-coagulated blood was collected with written informed consent from women at risk of ALD or from known ALD heterozygote women who were participants in a Lorenzo’s oil trial. Controls included women who tested negative for an *ABCD1* gene mutation and males who had normal plasma VLCFA in the Peroxisomal Laboratory at the Kennedy Krieger Institute. DBS were made from a portion of the anonymised whole blood that had been collected for diagnostic testing.

**Supplemental Methods 1:**

C26:0-Lysophosphatidylcholine (C26:0-LPC), is the metabolite found to be increased in males and females with ALD. Measurement of C26:0-LPC in the Peroxisomal Laboratory at the Kennedy Krieger Institute, Baltimore, MD is performed by LC-MS/MS. For postnatal diagnosis, whole blood anti-coagulated is spotted on filter paper used for newborn screening, such as Whatman # 10534612 protein saver 903 cards. The blood is air dried and stored at -25^0^C. until analysis.

For quality control, DBS with known amounts of C26:0-LPC from the Center For Disease Control, and DBS from 1 control, 1 male with ALD and 1 woman with ALD were assayed with each set of diagnostic analyses. To the 1/8” punched DBS in a 13 x 100mm glass test tube, or in a 96-well plate, 150 µl of methanol solution containing

15pmoles of the internal standard ^2^H_4_-26:0-LPC was added. Samples were mixed and placed on a shaker at room temperature for 25 min and then centrifuged at 800G. The 150 µl supernatant was transferred to an injection vial or 96-well plate for measurement of C26:0-LPC. The C26:0-LPC was analyzed on an AB SCIEX API 3200 mass spectrometer interfaced with a Shimadzu UFLC liquid chromatograph. A volume of 8 µl of sample was injected for combined liquid chromatography-tandem mass spectrometric (LC-MS/MS) analysis using a Waters X-Terra C8 column (2.1mm x 50 mm x 3.5u) for chromatographic resolution of the analytes and internal standard extracted from the sample matrix. A gradient starting at injection with 75% mobile phase A comprised of H_2_O:CH_3_CN: HCOOH (54.5:45:0.5% containing 2mM NH_4_HCOOH) and 25% mobile phase B comprised of CHCl_3_:CH_3_CN: HCOOH (10:90:0.5% containing 2mM NH_4_HCOOH) to 100% mobile phase B at 1.0 minutes to 2.0 minutes post injection time at flow rate of 0.5ml/minute. The total run time for each sample injection, including the return to 25% mobile phase B at 2.5 minutes and a 0.5-minute equilibration time, is 3 minutes. The MRM transitions monitored were m/z 636 to m/z 104 and m/z 640 to m/z 104 for C26:0-LPC and ^2^H_4_-26:0-LPC respectively. Quantitation of C26:0 -LPC was performed by analyzing peak areas of the target analyte and its internal standard. Data from calibration curve for C26:0 -LPC shows a linear response (R^2^= 0.99) in a dynamic range of 0.1-20 pmoles per 1/8” DBS (Hubbard et al., 2009).

Statistical analyses were performed using the statistics package in R.

**Reagents:** High-purity grade HPLC solvents were obtained from the J.T. Baker Co or Burdick & Jackson, Inc. The stable isotope, ^2^H_4_-26:0-LPC and reference standards, C26:0-LPC and C24:0-LPC were purchased from Avanti Polar Lipids, Inc.
